# A New Missing Data Imputation Algorithm Applied to Electrical Data Loggers

**DOI:** 10.3390/s151229842

**Published:** 2015-12-10

**Authors:** Concepción Crespo Turrado, Fernando Sánchez Lasheras, José Luis Calvo-Rollé, Andrés José Piñón-Pazos, Francisco Javier de Cos Juez

**Affiliations:** 1 Maintenance Department, University of Oviedo, San Francisco 3, Oviedo 33007, Spain; ccrespo@uniovi.es; 2 Department of Construction and Manufacturing Engineering, University of Oviedo, Campus de Viesques, Gijón 33204, Spain; 3 Departamento de Ingeniería Industrial, University of A Coruña, A Coruña 15405, Spain; jlcalvo@udc.es (J.L.C.-R.), andres.pinon@udc.es (A.J.P.-P.); 4 Prospecting and Exploitation of Mines Department, University of Oviedo, Oviedo 33004, Spain; fjcos@uniovi.es

**Keywords:** missing data imputation, multivariate imputation by chained equations (MICE), Multivariate adaptive regression splines (MARS), quality of electric supply, voltage, current, power factor

## Abstract

Nowadays, data collection is a key process in the study of electrical power networks when searching for harmonics and a lack of balance among phases. In this context, the lack of data of any of the main electrical variables (phase-to-neutral voltage, phase-to-phase voltage, and current in each phase and power factor) adversely affects any time series study performed. When this occurs, a data imputation process must be accomplished in order to substitute the data that is missing for estimated values. This paper presents a novel missing data imputation method based on multivariate adaptive regression splines (MARS) and compares it with the well-known technique called multivariate imputation by chained equations (MICE). The results obtained demonstrate how the proposed method outperforms the MICE algorithm.

## 1. Introduction

The presence of harmonics in an electrical system is associated with many problems in its performance. The main problems are overheating in conductors, especially in the neutral ones, due to the skin effect, and activating automatic breakers producing problems with supply continuity. Finally, the deterioration of the waveform of the voltage harmonic distortion associated would cause malfunctions of some devices.

As the existence of harmonics cannot be avoided, monitoring in real-time is necessary in order to control them within certain limits. Additionally, sometimes they can be transferred by acting on the installation in order to avoid its effects by means of filters either active or passive. In these cases, the use of isolation transformers, super-immunized differential breakers, *etc*., must be studied.

Another problem frequently encountered in an electrical installation is the imbalance between phases. Although it is well known that balance is achieved by working at the highest levels of the installed capacity in order to take full advantage of the installation, sometimes this is not possible. An imbalance is usually caused by a bad load distribution between phases and provokes a high current return displayed by the neutral, as it has to compensate for the gap being at the center of the scheme vectors. These problems will increase if these charges are also producing linear and harmonic distortion. In addition, imbalances may also cause the performance of the protection of the low voltage at the output of the transformer arise above its caliber in the overloaded phase currents.

In this context, the quality of electricity is a problem represented in all of its parameters: voltage, current, frequency anomalies, *etc*., that cause failures or disability of electrical or electronic devices [1]. Nowadays, the quality of electricity is a challenge in terms of efficiency, optimization, stability, fault prevention, and so on [2]. Science and technology have advanced, and continue to do so, significantly, with the aim of mitigating some of the consequent typical problems, which disturb electrical quality and, thus, the above mentioned challenges would be impossible to overcome, at least satisfactorily [3].

There are several different contributions with the above-mentioned aim. For example, in [4] a new power quality deviation index based on principal curves is proposed. [5] shows a complete review of signal processing and intelligent methods used for self-classification of power quality events and an influence of noise on recognition and classification of perturbations. A smart instrument used for recognition, labeling, and quantitation of power and energy quality disturbance is described in [6]. In the same way, [7] presents an intelligent instrument for instantaneous high-resolution frequency measurement in accordance with typical indicator values for the quality of electrical power control and monitoring, while [8] describes a communication infrastructure developed to obtain reliable data delivery with low cost, in order to avoid the problems in the provision of the power quality monitoring service.

In some buildings it would be of interest to monitor the main electrical parameters. This real-time monitoring and control is required in order to balance new loads and reduce the general consumption of the building by means of the assessment of the residual consumption (or consumption out of working hours). Such information is also useful to optimize the rates to be contracted. Additionally, this monitoring would be useful for studying supply problems due to lack of balance or harmonics, for analyzing the quality of the energy, and also for preventing incidents with the machinery due to poor signal quality. Finally, it would also be of interest to study the operation of the building and analyze its efficiency depending on parameters such us the number of people who use it, power installed, square meters in use, *etc.*

During the data collection process it is possible, due to different circumstances, for a small amount of the information retrieved to be lost. For these situations it is important to have missing data imputation. A process of missing data imputation consist of filling missing values in data series with estimated ones.

The quality of the electric supply of buildings is not only limited to the continuity of the supply, as concepts such as reliability, safety, and maintenance are also important indicators. It is also necessary that the available information be complete. The lack of information in some records, generally translated as zeros, distort the results.

There is also an important economic component of the data record, and that is the optimization of supply contracts, in other words, knowing the consumption of a building distributed over time. It is possible to associate the activities in the building so that we obtain a balanced installation, performing the most demanding activities at the most convenient hours of the day. To this end, it is necessary to collect information both before the decision and after the implementation of measures, in order to compare similar periods of expenditure. The latter would also serve in the event that energy-saving measures of another kind, such as replacing lighting by low consumption, placing detectors in corridors, placing inverters in circulation pumps, *etc*., were implemented.

This paper evaluates a new imputation method, which allows the system to fill in the missing data of any of the sensor devices that are used in this research for the recording of voltages, currents and power factors. The proposed algorithm is based on multivariate adaptive regression splines and outperforms the results obtained by a benchmark method, as it is the multivariate imputation by chained equations (MICE) [9].

Nowadays, the two major methods for missing data imputation are multiple imputation and maximum likelihood. The maximum likelihood chooses as parameter estimates those values which, if true, would maximize the probability that have in fact been observed. The multiple imputation is based on different methodologies but all follow these steps: some random variation is introduced into the data set and several imputed data sets are generated. After that, those data sets are used for problem analysis and finally the combination of the results into a single set of parameter estimates, standard errors and test statistics is made. Since the missing at random (MAR) assumption cannot be checked from the data at hand, it is important to take into account if missing data can be considered as MAR. In those cases that cannot be considered, they called not missing at random (NMAR). Several models of NMAR data have been developed and its detailed analysis is beyond the scope of the present research.

The rest of the paper is organized as follows: Section 2 includes information about the measurement equipment employed and a description of the data recorded. Section 3 describes the new proposed algorithm and the benchmark technique employed detailing also the two metrics used for comparing their performance. A comparison of the results achieved with each method for different levels of missing data is presented in Section 4. Finally, conclusions are drawn in Section 5.

## 2. Experimental Section

### 2.1. Measurement Equipment

In the present research, the devices employed are specific to the measurement of power quality variables, which are described in this section. They have some common measurement features in common, namely: Voltage Line/Neutral (V. L/N), Voltage Line/Line (V. L/L), Current by line (Current), Power Input/Output (+/− Watts), Energy Input/Output (+/− Wh), Reactive Power (+/− VARs), Reactive Power Input/Output (+/− VARh), Apparent Power (VA), Apparent Energy (VAh), Power Factor (PF), and Frequency (Frequency). Table 1 shows the accuracy for each device during the different electrical measurements. It should be noted that the values shown in percentage corresponds to the reading percentage.

**Table 1:** The variables accuracy for each device.

Variable	Units	S-100	S200	NEXUS 1252		MP200
				200 ms	1 s	
**V. L/N**	V, KV	0.1%	0.1%	0.1%	0.05%	0.3%
**V. L/L**	V, KV	0.1%	0.2%	0.1%	0.05%	0.5%
**Current**	A, KA	0.1%	0.1%	0.1%	0.025%	0.3%
**+/− watts**	W	0.2%	0.2%	0.1%	0.06%	0.5%
**+/− wh**	Wh	0.2%	0.2%	N/A	0.04%	0.5%
**+/− VARs**	VARs	0.2%	0.2%	0.1%	0.08%	1.0%
**+/− VARh**	VARh	0.2%	0.2%	N/A	0.08%	1.0%
**VA**	VA	0.2%	0.2%	0.1%	0.1%	1.0%
**VAh**	VAh	0.2%	0.2%	N/A	0.08%	1.0%
**FP**	+/−0.5 to 1	0.2%	0.2%	0.1%	0.08%	1.0%
**Frequency**	Hertz	1.10^-2^	+/−3.10^-2^	3.10^-2^	1.10^-2^	+/−1.10^-2^

The four devices used in the present study can perform all the mentioned measurements [10]; also, each device has additional capabilities that are discussed below in the following subsections.

### 2.2. Shark 100 (S-100)

One of the options included for this equipment is the optical IrDA port, which allows the programing of the device from a laptop or personal digital assistant (PDA). Additionally, it incorporates V-Switch technology. This tool lets the users update and include the required functions using programing commands, even after the installation of the device installation. The offered VSwitches (VSw) offered are:

• VSw 1—Volts and Amperes Meter—Default.

• VSw 2—Volts, Amperes, kW, kVA, kVAR, Frequency, PF.

• VSw 3—Volts, Amperes, kW, kVA, kVAR, Frequency, PF, kVAh, kVARh, kWh and Distributed Network Protocol (DNP) v.3.0.

• VSw 4—Volts, Amperes, kW, kVA, kVAR, Frequency, PF, kVAh, kVARh, kWh, %THD (total harmonic distortion), Boundary Alerts and Distributed Network Protocol (DNP) v.3.0.

A RS485 Port can be added as an option. With it, communication is feasible by using Modbus or DNP 3.0 Protocols. In addition to the RS485, the device also incorporates a KYZ pulse, which is used to send instantaneous information regarding energy consumption to other devices. It is possible to add an Ethernet option with the INP10 module, which is a 10/100BaseT Ethernet with the Modbus TCP protocol.

### 2.3. Shark 200 (S-200)

The Shark 200 system is a small-size device used for power and energy measurements. It provides an invoicing measuring feature, in conjunction with an advanced data recording system, measurement of the electrical power quality, communication, and I/O capabilities. This equipment also includes V-Switch technology. The V-Switches in this case incorporate the features shown in the Table 2.

**Table 2:** Features of the V-Switches technology.

Feature	Vs1	Vs2	Vs3	Vs4	Vs5	Vs6
Input/Output Expansion and Multifunction Measurement	√	√	√	√	√	√
2 MB (Megabytes) datalogging (dl)		√	√	√		
3 MB -dl					√	
4 MB -dl						√
Harmonic Study			√	√	√	√
TLC (transformers line compensation) and CT (Current transformers) / PT (Power Current) Compensation	√	√	√	√	√	√
Functions for Control and Limits Configuration				√	√	√
64 SPC (samples per cycle) Waves Datalogger					√	
512 SPC Waves Datalogger						√

The Shark 200 device from feature V2 to V6, offers the possibility of data recording by using historic tendencies, limit alerts, input/output deviations, and events categorization. For the V5 and V6 models, the waveform can be recorded.

It is possible to make an independent CBEMA (Computer and Business Equipment Manufacturers Association’s) log plotter: The system records an independent CBEMA and it makes an autonomous CBEMA record for size, as well as potential event times.

The S-200 model offers an on-line harmonic analysis from to the 40th up to the 255th order for current and voltage inputs.

Regarding communication, this model includes the following features:

• One port RS485 port allows communication using Distributed Network Protocol (DNP) v.3.0 or Modbus protocols.
• KYZ Pulse—this device incorporates Pulse Outputs mapped to total energy.
• Furthermore, it has an optical IrDA port with the same functions as the previously-explained model.

### 2.4. Nexus 1252

In general terms, this device has advanced features that offer a global view of power and energy usage and, of course, visualization of the quality of electrical power within a power network. The device is able to capture a maximum of 512 samples per working cycle by event. Additionally, this device performs events analysis by 16 bits A/D converter, for electric voltage and electric current, which offers high-resolution. Furthermore, it is possible to activate a waveform datalogging by triggers that enable power quality surveys, fault detection, and the like, to be performed.

In terms of harmonic measurements, the device is capable of measuring up to the 255th order, in the case of current and voltage. If necessary, it can measure the harmonics in real time up to the 128th order. The device provides the THD percentage and the K-Factor with the harmonics. Additionally, it is possible to monitor switching noise from several elements of an installation. Like the previous device, the Nexus 1252 is able to make an independent CBEMA, and it makes an autonomous CBEMA log for size and time of potential events, which gives the consequent advantages mentioned previously.

In terms of communications, the device has four ports, and each one is able to communicate in several common protocols, with the aim of reading purposes and control simultaneously. Several peripherals are available for displaying or for external I/O options.

### 2.5. Shark MP200

The MP200 model measures and provides information of power usage from eight three-phase WYE circuits or from twenty-four single-phase systems. The MP200 system can create precise reports of power usage, analyze peak demand, and provide control signals to limit peak demand and billing based on usage and demand.

The MP200 offers communication possibilities like the previous models. One typical USB port and two standard RS485 ports, with optional RJ45 wired, or 802.11 WiFi, are provided. These ports support standard protocols as Modbus ASCII, RTU, and TCP/IP. By V-Switch options, the MP200 can be configured for basic sensors with real-time data (V1) to Advanced Logger up to 2400 Days (V3).

### 2.6. Description of the Data

The data set employed for the present research corresponds to measurements of the voltage phase to neutrum, (three variables) phase-to-phase voltage (three variables), current in each phase (three variables) and the average power factor (one variable) of a three-phase electrical supply of a building. The records were taken each 15 min from 27 November 2014 at 18:45 to 31 May 2015 at 23:45.

The building under study in the present research belongs to the University of Oviedo (Spain). This building is called Severo Ochoa after the Nobel Prize-winning scientist and has five floors and two basement levels that sum a total of 8150 m^2^. This building holds the Information Technology Services of the University including their server rooms and some scientific laboratories that include equipment such as nuclear magnetic resonance spectrometers, electron microscopes, X-ray diffractometers, and the like. For all these kind of facilities it is essential to guarantee a good quality standard of electrical supply 24 h a day, every day of the week. A total of 78 employees work in this building, which has an average daily energy consumption of 190,572 kWh.

### 2.7. Harmonics and Harmonic Distortion

The large number of heterogeneous receivers in the building, such as computers, uninterruptible power supply devices, ballasts of fluorescent lighting systems, variable speed drives, induction ovens, and capacitors all create harmonic distortions in the net. All of these non-linear loads cause the flow of harmonic currents in the distribution system.

According to Fourier’s theorem, a periodic continuous function f(x) with a period of 2·L may be expressed as the sum of a series of sine or cosine terms each of which has a specific amplitude and phase coefficients known as Fourier coefficients. This theorem can be expressed with the following formula [11]:

(1) $$\text{f}(\text{x})=\frac{1}{2}\cdot a_{0}+\sum_{n=1}^{\infty }\left[a_{n}\cdot \cos\left(\frac{n\cdot \pi \cdot x}{L}\right)+b_{n}\cdot \sin\left(\frac{n\cdot \pi \cdot x}{L}\right)\right]$$

where:

(2) $$a_{n}=\frac{1}{L}\int_{-L}^{L}f\left(x\right)\cdot \cos\left(\frac{n\cdot \pi \cdot x}{L}\right)\cdot dx$$

(3) $$b_{n}=\frac{1}{L}\int_{-L}^{L}f\left(x\right)\cdot \text{sin}\left(\frac{n\cdot \pi \cdot x}{L}\right)\cdot dx$$

Harmonic frequencies are multiples of the waveform’s fundamental frequency. The harmonic distortion may be defined as the degree to which a waveform deviates from a pure sinusoidal wave. In the case of an ideal sine wave, its harmonic component is equal to zero. The total harmonic distortion (THD) is defined as the sum of all harmonic components of the voltage or current waveform compared to the fundamental component of the voltage or current wave. For the case of the current, the THD formula can be expressed as follows:

(4) $$THD=\frac{\sqrt{\sum_{i=1}^{\infty }I_{i}^{2}}}{I_{1}}$$

where $I_{i}$ represents the amplitude of the different harmonics.

## 3. Methodology

The data set is made up of a total of 17,763 samples that correspond to the period of time referred in the description of the data. It is used to test two different algorithms: multiple imputation by chained equations (MICE) and the proposed algorithm AAA (Adaptive Assignation Algorithm). The dataset is submitted to a process of random data deletion. This process consisted of supposing that the probability of an observation being missing does not depend on observed or unobserved measurements. It is called missing-completely-at-random (MCAR). The process of random data deletion was repeated five times for three different levels of missing data: 10%, 15%, and 20% of the total. After each deletion process, both algorithms were applied to the resulting data subset and the performance of the two methods compared.

### 3.1. Multivariate Adaptive Regression Splines (MARS)

The algorithm proposed in the present research is based on the computation of multivariate adaptive regression splines (MARS) models, for the prediction of the missing values. MARS is a multivariate nonparametric technique [12]. Its main purpose is to predict the values of a continuous dependent variable, y (n×1), from a set of independent exploratory variables, X (n×p). This model can be represented by the following Equation [13,14]:

(5) y=f(X)+e

where f is a balanced sum of basis functions that depend on X and e is the error vector. One of the main advantages of MARS models is that they do not require any *a priori* assumptions about the functional relationships between dependent and independent variables [15,16,17]. The reason is that this relation is driven by the basis function determined by the regression data (X,y).

MARS is a generalization of classification and regression trees [18] and is able to overcome some of the limitation of this method. The MARS regression model is constructed by means of basis functions called splines. These splines are defined as follows:

(6) $${\left[-\left(x-t\right)\right]}_{+}^{q}=\{\begin{array}{c} {\left(t-x\right)}^{q}\;\;\;\;\;\;\;\;if\;x<t\\ 0\;\;\;\;\;\;\;\;\;\;\;\;\;\;\;\;\;\;\;\;\;\;otherwise \end{array}$$

(7) $${\left[-\left(x-t\right)\right]}_{+}^{q}=\{\begin{array}{c} {\left(t-x\right)}^{q}\;\;\;\;\;\;\;\;if\;x<t\\ 0\;\;\;\;\;\;\;\;\;\;\;\;\;\;\;\;\;\;\;\;\;\;otherwise \end{array}$$

### 3.2. The Proposed Algorithm AAA

In order to introduce the new algorithm, let us assume that we have a dataset formed by n different variables $v_{1},\;v_{2},\;\ldots ,\;v_{n}$. In order to calculate the missing values of the i-th column, all the rows with no missing value in the said column are employed. Then, a certain number of MARS models are calculated. It is possible to find rows with very different amounts of missing data from zero (no missing data) to n (all values are missing). Those columns with all values missing will be removed and will be neither used for the model calculation nor imputed. Therefore, any amount of missing data from 0 to n−2 is feasible (all variables but one with missing values).

In other words, if the dataset is formed by variables $v_{1},\;v_{2},\;\ldots ,\;v_{n}$. and we want to estimate the missing values in column $v_{i}$, then the maximum number of different MARS models that would be computed for this variable (and in general for each column) is as follows: $\sum_{k=1}^{n-1}\left(\begin{array}{c} n-1\\ k \end{array}\right)$. For the case of the data under study in this research, with 10 different variables, a maximum of 5110 distinct MARS models would be trained (511 for each variable).

Table 3 represents the 25 first rows of the dataset in which the algorithm will be applied. When the algorithm is applied to the third column of these datasets (variable $v_{3}$), all those rows with missing data (represented by means of the symbol ‘o’) in the third column are not employed for the calculus of the models (rows in red). If those rows were removed, different models would be trained for the prediction of $v_{3}$ using different subsets of variables. Continuing with the example of variable $v_{3}$ and taking into account the data missing in the 25 first rows, it would be possible to train the following models:

Model 1: a model that uses as output variable $v_{3}$ and the other nine as input variables ($v_{1},\;v_{2},\;v_{4},\;v_{5},\;v_{6},\;v_{7},\;v_{8},\;v_{9},\;v_{10}$).

Model 2: a model that uses as output variable $v_{3}$ and as input variables $v_{2},\;v_{4},\;v_{5},\;v_{6},\;v_{7},\;v_{8},\;v_{9},\;v_{10}$.

Model 3: a model that uses as output variable $v_{3}$ and as input variables $v_{1},\;\;v_{4},\;v_{5},\;v_{6},\;v_{7},\;v_{8},\;v_{9},\;v_{10}$.

Model 4: a model that uses as output variable $v_{3}$ and as input variables $v_{1},\;v_{2},\;v_{4},\;v_{6},\;v_{7},\;v_{8},\;v_{9},\;v_{10}$.

Model 5: a model that uses as output variable $v_{3}$ and as input variables $v_{1},\;v_{2},\;v_{4},\;v_{5},\;v_{7},\;v_{8},\;v_{9},\;v_{10}$.

Model 6: a model that uses as output variable $v_{3}$ and as input variables $v_{4},\;v_{5},\;v_{6},\;\;v_{7},\;v_{8},\;v_{9},\;v_{10}$.

Model 7: a model that uses as output variable $v_{3}$ and as input variables $v_{1},\;v_{5},\;v_{6},\;v_{7},\;v_{8},\;v_{9},\;v_{10}$.

Model 8: a model that uses as output variable $v_{3}$ and as input variables $v_{1},\;v_{4},\;v_{5},\;v_{6},\;v_{8},\;v_{9},\;v_{10}$.

After the calculation of all the available models, the missing data of each row will be calculated using those models that employ all the available non-missing variables of the row. In those cases in which no model was calculated, the missing data will be replaced by the median of the column. Please note in that the case of large data sets with a not-too-high percentage of missing data, these will be an infrequent case. In the case of missing completely at random data, the probability, represented by letter *Q*, of not having at least two non-missing values in a certain row can be expressed by the following formula:

(8) $$Q=p^{n}+\left(1-p\right)\cdot \left(n-1\right)\cdot p^{n-1}$$

where: *N* is the number of variables; *P* is the rate of missing data in a MCAR case.

In the case of our example, none of the rows was in this situation for the 10 and 15% of missing data, while in the case of 20% of missing data it happened only in one line (less than 0.006% of the total amount of lines). These results are in line with those expected by the formula.

As a general rule for the algorithm, it has been decided that when certain value can be estimated using more than one MARS model, it must be estimated using the MARS model with the largest number of input variables; the value would be estimated by any of those models chosen at random. Finally, in those exceptional cases in which no model is available for estimation, the median value of the variable will be used for the imputation.

**Table 3:** Example of the dataset (25 first rows).

Row #	v_1_	v_2_	v_3_	v_4_	v_5_	v_6_	v_7_	v_8_	v_9_	v_10_	Model 1	Model 2	Model 3	Model 4	Model 5	Model 6	Model 7	Model 8
1	X	X	X	X	X	X	X	X	X	X	Yes	yes	yes	yes	yes	yes	yes	yes
2	X	o	X	o	X	X	X	X	X	X	No	no	yes	no	no	yes	yes	no
3	X	X	X	X	o	X	X	X	X	X	No	no	no	yes	no	no	no	no
4	X	X	o	o	X	X	X	X	X	X	No	no	no	no	no	no	no	no
5	X	X	X	X	X	X	X	X	X	X	Yes	yes	yes	yes	yes	yes	yes	yes
6	X	o	X	X	X	X	X	X	X	X	No	no	yes	no	no	yes	yes	yes
7	o	X	X	X	X	X	X	X	X	X	No	yes	no	no	no	yes	no	no
8	X	X	X	X	X	X	X	X	X	X	Yes	yes	yes	yes	yes	yes	yes	yes
9	o	o	o	X	X	X	X	X	X	X	No	no	no	no	no	no	no	no
10	X	X	o	X	X	X	X	X	X	X	No	no	no	no	no	no	no	no
11	X	X	o	X	X	X	X	X	X	X	No	no	no	no	no	no	no	no
12	X	o	o	X	X	X	X	X	X	X	No	no	no	no	no	no	no	no
13	X	X	X	X	X	X	X	X	X	X	Yes	yes	yes	yes	yes	yes	yes	yes
14	o	o	X	X	X	X	X	X	X	X	No	no	no	no	no	yes	no	no
15	o	X	X	X	X	X	X	X	X	X	No	yes	no	no	no	yes	no	no
16	X	X	X	X	X	X	X	X	X	X	Yes	yes	yes	yes	yes	yes	yes	yes
17	o	o	o	o	o	o	X	X	X	X	No	no	no	no	no	no	no	no
18	X	X	X	X	X	X	X	X	X	X	Yes	yes	yes	yes	yes	yes	yes	yes
19	X	o	X	X	X	X	o	X	X	X	No	no	yes	no	no	no	no	yes
20	X	X	X	X	X	o	X	X	X	X	No	no	no	no	yes	no	no	no
21	X	o	o	X	o	X	X	X	X	X	No	no	no	no	no	no	no	no
22	X	X	X	X	X	X	X	X	X	X	Yes	yes	yes	yes	yes	yes	yes	yes
23	X	X	o	o	X	X	X	X	X	X	No	no	no	no	no	no	no	no
24	X	X	X	X	X	o	X	X	X	X	No	no	no	no	yes	no	no	no
25	X	X	o	X	X	X	X	X	o	X	No	no	no	no	no	no	no	no

### 3.3. The Benchmark Rechnique: The MICE Algorithm

The algorithm called multiple imputation by chained equations (MICE) algorithm was developed by van Buuren and Groothuis-Oudshoorn [19]. This referred algorithm is a Markov Chain Monte Carlo Method in which the state space is the collection of all imputed values [9]. As with any other Markov Chain, the MICE algorithm has to accomplish three properties [20,21,22,23] in order to converge. The referred properties are as follows:

The chain must be able to reach all parts of the state space. This means that it is irreducible.

The chain should not oscillate between different states. In other words, the Markov Chain must be aperiodic.

Finally, the chain must be recurrent. This means, as in any other Markov Chain, that the probability of the chain of starting from i and returning to i will be equal to one.

According to the experience of the algorithm creator [19], and also from our own previous experience [9], the convergence of the MICE algorithm is achieved after a relatively low number of iterations, usually somewhere between five and 20 [23]. In the case of the present research, up to 20 iterations were considered but as not statistically significant improvements with respect to five iterations were achieved, the results for five iterations are presented.

The MICE algorithm [23] for the imputation of multivariate missing data consists of the steps that are listed in Algorithm 1. In this algorithm Y represents a n×p matrix of partially-observed sample data, R is a n×p matrix, 0−1 response indicators of Y, and ∅ represents the parameters space. This methodology was already explained by the authors in previous research published in this journal [9]. For a more detailed explanation of the algorithm we recommend another look at the original research by van Buuren and Groothuis-Oudshoorn [23].

**Algorithm 1.** MICE algorithm for imputation of multivariate missing data [19].
Specify an imputation model $P(Y_{j}^{mis}|Y_{j}^{obs},Y_{-j},R)$ for variable $Y_{j}$ with j=1,…,p.For each j, fill in starting imputations $Y_{j}^{0}$ by random draws from $Y_{j}^{obs}$.Repeat for t=1,…,T (iterations).Repeat for j=1,…,p (variables).Define $Y_{-j}^{t}=\left(Y_{1}^{t},\ldots ,Y_{j-1}^{t},Y_{j+1}^{t-1},\ldots ,Y_{p}^{t-1}\right)$ as the currently complete data except $Y_{j}$.Draw $\emptyset_{j}^{t}\sim P\left(\emptyset_{j}^{t}\text{|}Y_{j}^{obs},Y_{-j}^{t},R\right).$Draw imputations $Y_{j}^{t}\sim P\left(Y_{j}^{mis}\text{|}Y_{j}^{obs},Y_{-j}^{t},R,\emptyset_{j}^{t}\right)$.End repeat j.End repeat t.

### 3.4. Performance of the Algorithms

The performance of the proposed algorithm in comparison with MICE has been evaluated using the mean absolute error (MAE) and the root mean square error (RMSE). MAE measures the average magnitude of the error in a set of forecasts without considering their direction. It is a linear score, which weights all the individual differences equally, while RMSE is a quadratic scoring rule, which measures the average magnitude of the error. In the case of the RMSE, as errors are squared before they are averaged, it gives a relatively higher weight to large errors. When results are analyzed using both variables, it should be noted that the greater the difference between them, the greater the variance in the individual errors in the sample, taking into account that the lower their values, the better the model.

The formulae for both kind of errors are as follows:

(9) $$MAE=\frac{1}{n}\cdot \sum_{i=1}^{n}\left|e_{i}\right|$$

(10) $$RMSE=\sqrt{\frac{1}{n}\cdot \sum_{i=1}^{n}e_{i}^{2}}$$

where: n is the number of samples; $e_{i}$ is the error of the i-th sample calculated as the difference of predicted value versus real value.

The present article uses both RMSE and MAE. The underlying assumption when presenting RMSE [24] is that the errors are unbiased and follow a normal distribution. The MAE is suitable to describe uniformly distributed errors. As model errors are likely to have a normal distribution, the RMSE is a better metric to present than the MAE for such kind of data. Although in the case of errors following a normal distribution RMSE is more appropriate to use than MAE, it is the preferred metric for the indication of the model average error.

## 4. Results and Discussion

In this section the results of the MICE algorithm and the proposed one AAA package are presented and their performances compared. As was already stated in the section describing the data, due to the random component of both algorithms, a process of MCAR data deletion of 10%, 15%, and 20% of the information was performed five times. The performance of both algorithms was compared by means of RMSE and MAE metrics. In order to verify that the results obtained with the proposed AAA package for the five different iterations were better than those achieved by other methods, the results of the five iterations are presented. Those tables also contain the average values of the five replications the iterations with the same number use the same database. Table 4 shows the RMSE values of the MICE and the proposed AAA package when applied to a database with 10% of the data missing. As can be observed for the ten variables considered, the RMSE values obtained by the new algorithm are considerably lower than those obtained using the MICE method. On average they are 15 times lower, and in all cases the RMSE values of the proposed algorithm are considerably lower. The results obtained for missing data rates of 15% (Table 5) and 20% (Table 6) are similar to those obtained for the missing rates of 10%.

**Table 4:** RMSE obtained with a 10% of missing data MICE and new proposed AAA package.

	RMSE MICE 10% MISSING DATA									
Iteration	Van	Vbn	Vcn	Vab	Vbc	Vca	Ia	Ib	Ic	PF
1	19.6804	33.2038	20.8058	37.7042	39.6212	48.5060	0.2294	0.2501	1.8328	0.0031
2	17.5901	30.4712	22.9721	41.6324	28.1667	49.4147	0.3048	0.3419	1.7036	0.0032
3	17.7238	29.2717	22.8719	32.0352	37.9528	49.5132	0.2612	0.3322	1.8113	0.0030
4	16.1665	30.9164	20.1289	41.8040	28.4577	47.6496	0.2710	0.2344	1.8349	0.0033
5	18.8739	33.3065	20.9843	32.6096	40.9730	45.3324	0.2492	0.2768	1.6502	0.0031
Average	18.0069	31.4339	21.5526	37.1571	35.0343	48.0832	0.2631	0.2871	1.7666	0.0032
	**RMSE NEW ALGORITHM 10% MISSING DATA**									
**Iteration**	**Van**	**Vbn**	**Vcn**	**Vab**	**Vbc**	**Vca**	**Ia**	**Ib**	**Ic**	**PF**
1	1.5556	1.6047	0.9758	1.5621	2.1820	1.8054	0.1320	0.1446	0.1233	0.0020
2	1.1417	1.0847	1.0334	1.5623	2.0075	1.8990	0.1441	0.1397	0.1206	0.0020
3	1.0186	1.0077	0.8325	2.6758	1.6684	1.8550	0.1366	0.1458	0.1170	0.0020
4	1.0750	1.1247	1.1410	1.4569	1.7598	1.6958	0.1349	0.1558	0.1278	0.0017
5	1.1056	1.0992	0.9680	1.6783	1.9487	1.8209	0.1331	0.1317	0.1128	0.0021
Average	1.1793	1.1842	0.9901	1.7871	1.9133	1.8152	0.1361	0.1435	0.1203	0.0020

**Table 5:** RMSE obtained with a 15% of missing data MICE and new proposed AAA package.

	RMSE MICE 15% MISSING DATA									
**Iteration**	**Van**	**Vbn**	**Vcn**	**Vab**	**Vbc**	**Vca**	**Ia**	**Ib**	**Ic**	**PF**
1	17.0837	28.8975	23.1269	38.2302	26.7259	45.7781	0.3816	0.2352	1.8578	0.0031
2	19.7831	31.6292	21.6406	44.4176	31.1867	50.5978	0.2733	0.4289	1.6515	0.0030
3	16.8887	32.1573	23.2565	34.8709	36.4404	49.8771	2.0080	0.3768	0.4198	0.0032
4	18.9432	30.8065	21.1655	43.2729	32.0558	43.2723	0.3458	0.3326	1.7407	0.0028
5	19.0647	30.0262	23.5861	32.4402	28.9609	44.9738	0.5376	0.2517	1.8402	0.0034
Average	18.3527	30.7033	22.5551	38.6463	31.0739	46.8998	0.7092	0.3251	1.5020	0.0031
	**RMSE NEW ALGORITHM 15% MISSING DATA**									
**Iteration**	**Van**	**Vbn**	**Vcn**	**Vab**	**Vbc**	**Vca**	**Ia**	**Ib**	**Ic**	**PF**
1	1.0916	1.0625	0.9937	1.6562	1.7851	1.7874	0.1355	0.1314	0.1184	0.0021
2	1.1417	1.0061	0.9990	1.6799	1.9229	1.7843	0.1285	0.1446	0.1235	0.0019
3	1.1178	1.1311	1.0816	1.7174	2.3560	1.7111	0.1345	0.1460	0.1249	0.0021
4	1.5109	1.0043	1.1689	1.5117	1.9786	1.8057	0.1250	0.1394	0.1262	0.0018
5	1.1151	1.0109	1.0351	1.6381	2.5543	1.8637	0.1290	0.1364	0.1324	0.0019
Average	1.1954	1.0430	1.0556	1.6406	2.1194	1.7904	0.1305	0.1396	0.1250	0.0020

Something similar to the RMSE occurs with the values obtained for the MAE metric. In this case, also, the values obtained with the new AAA package are significantly lower than those obtained for the MICE algorithm in the three cases: 10% (Table 7), 15% (Table 8), and 20% (Table 9). Please also note that the average improvement of the MAE values in the case of the proposed algorithm is 15 times greater than the MAE values obtained by means of the MICE algorithm.

**Table 6:** RMSE obtained with a 20% of missing data MICE and new proposed AAA package.

	RMSE MICE 20% MISSING DATA									
Iteration	Van	Vbn	Vcn	Vab	Vbc	Vca	Ia	Ib	Ic	PF
1	16.5536	31.8986	22.5109	40.5584	32.8036	45.9128	0.2721	0.3250	1.8791	0.0032
2	18.5886	33.8541	23.3965	37.1860	28.7115	45.3711	1.9242	0.3857	0.4218	0.0031
3	18.0006	27.0257	22.4172	45.9451	29.7499	46.9085	0.4640	0.4657	1.8450	0.0031
4	18.4734	33.6455	22.6390	34.8455	42.4674	48.5727	0.2675	0.2951	1.6894	0.0032
5	19.0185	31.5739	22.9867	36.8936	30.8237	48.1872	0.2883	0.3618	1.8228	0.0029
Average	18.1269	31.5996	22.7900	39.0857	32.9112	46.9905	0.6432	0.3667	1.5316	0.0031
	**RMSE NEW ALGORITHM 20% MISSING DATA**									
**Iteration**	**Van**	**Vbn**	**Vcn**	**Vab**	**Vbc**	**Vca**	**Ia**	**Ib**	**Ic**	**PF**
1	1.0303	1.0031	1.0081	1.5567	1.7295	2.1989	0.1293	0.1535	0.1168	0.0018
2	1.3300	0.9645	1.4421	1.6901	1.9225	1.9970	0.1386	0.1430	0.1197	0.0019
3	1.4028	1.0751	1.0209	1.5444	1.6905	2.1216	0.1384	0.1462	0.1256	0.0018
4	1.1410	0.9442	0.9836	1.7262	1.8554	1.7554	0.1307	0.1391	0.1279	0.0019
5	1.0760	1.0285	1.0464	1.6981	1.8016	2.1696	0.1396	0.1351	0.1233	0.0019
Average	1.1960	1.0031	1.1003	1.6431	1.7999	2.0485	0.1353	0.1434	0.1226	0.0019

**Table 7:** MAE obtained with a 10% of missing data MICE and new proposed AAA package.

	MAE MICE 10% MISSING DATA									
Iteration	Van	Vbn	Vcn	Vab	Vbc	Vca	Ia	Ib	Ic	PF
1	15.3661	26.3015	16.4787	29.9178	30.6437	38.7431	0.1769	0.1910	1.3884	0.0026
2	13.4635	23.0373	19.0932	32.4891	23.0155	39.6558	0.2150	0.2234	1.2821	0.0026
3	13.9818	22.8360	18.2539	25.1375	29.5444	40.2728	0.2001	0.2107	1.3684	0.0024
4	12.9084	24.4469	16.2147	33.7170	22.3214	36.2061	0.2236	0.1892	1.3993	0.0026
5	14.9136	26.4011	16.7147	26.2535	32.1382	34.5887	0.1964	0.2052	1.2552	0.0025
Average	14.1266	24.6045	17.3510	29.5030	27.5326	37.8933	0.2024	0.2039	1.3387	0.0025
	**MAE NEW ALGORITHM 10% MISSING DATA**									
**Iteration**	**Van**	**Vbn**	**Vcn**	**Vab**	**Vbc**	**Vca**	**Ia**	**Ib**	**Ic**	**PF**
1	0.8954	0.8944	0.7444	1.1966	1.7099	1.4210	0.1053	0.1134	0.0971	0.0016
2	0.9017	0.8568	0.8358	1.2213	1.5561	1.5129	0.1120	0.1093	0.0960	0.0016
3	0.8236	0.7970	0.6533	1.4566	1.2741	1.4450	0.1123	0.1140	0.0881	0.0016
4	0.8617	0.8708	0.9240	1.1401	1.4034	1.3599	0.1045	0.1220	0.0975	0.0013
5	0.9135	0.8886	0.7926	1.3332	1.5911	1.4610	0.1057	0.1044	0.0920	0.0016
Average	0.8792	0.8615	0.7900	1.2696	1.5069	1.4400	0.1080	0.1126	0.0941	0.0016

For each of the ten variables involved in the present study, two-way ANOVA tests were performed in order to examine the influence of the kind of algorithm employed for the imputation (MICE versus proposed algorithm), the level of missing data (10%, 15% and 20%) and the interaction of both factors. These studies were carried out for the RMSE and MAE metrics. The influence of the model employed was found in all the variables for both metrics. Neither the percentage of missing data nor its interaction with the model employed for imputation were found to be significant in any of the variables. For the RMSE parameter the p-value was of p<0.001 for the kind of model (MICE vs proposed algorithm) in the variables $V_{an}$, $V_{bn}$, $V_{cn}$, $V_{ab}$, $V_{bc}$, $V_{ca}$, $I_{c}$ and PF, for $I_{a}$ was p=0.044 and for $I_{b}$ p=0.001. For the RMSE, when considering the variable percentage of missing data, there were no statistically significant differences between percentages (10, 15 and 20%) and the following p-values were obtained: 0.980 for $V_{an}$, 0.885 for $V_{bn}$ , 0.106 for $V_{cn}$, 0.921 for $V_{ab}$, 0.591 for $V_{bc}$ , 0.770 for $V_{ca}$ , 0.523 for $I_{a}$, 0.168 for $I_{b}$, 0.800 for $I_{c}$, and 0.784 for PF.

**Table 8:** MAE obtained with a 15% of missing data MICE and new proposed AAA package.

	MAE MICE 15% MISSING DATA									
Iteration	Van	Vbn	Vcn	Vab	Vbc	Vca	Ia	Ib	Ic	PF
1	12.7819	21.9118	18.6842	30.7929	21.5323	35.7718	0.2421	0.1845	1.4006	0.0024
2	15.6837	24.3796	17.1327	34.6552	24.3977	40.5020	0.1959	0.2489	1.3048	0.0023
3	12.9838	25.1110	18.3944	28.7176	28.7719	38.0755	1.5148	0.2157	0.2240	0.0025
4	14.3735	23.8986	17.4651	34.6752	25.7770	33.3585	0.2164	0.2117	1.3023	0.0022
5	14.9162	23.6717	18.2033	25.8131	23.0634	35.0113	0.2675	0.1877	1.4250	0.0026
Average	14.1478	23.7945	17.9759	30.9308	24.7084	36.5438	0.4873	0.2097	1.1314	0.0024
	**MAE NEW ALGORITHM 15% MISSING DATA**									
**Iteration**	**Van**	**Vbn**	**Vcn**	**Vab**	**Vbc**	**Vca**	**Ia**	**Ib**	**Ic**	**PF**
1	0.8882	0.8196	0.7866	1.3280	1.4423	1.3884	0.1091	0.1053	0.0973	0.0017
2	0.9152	0.8158	0.7646	1.3088	1.4749	1.3937	0.1035	0.1117	0.0986	0.0016
3	0.8710	0.9064	0.8255	1.3577	1.5212	1.3480	0.1067	0.1121	0.0961	0.0016
4	0.9951	0.7757	0.9129	1.2065	1.5648	1.4341	0.1002	0.1114	0.0983	0.0014
5	0.8625	0.7992	0.7959	1.2726	1.7787	1.4867	0.1029	0.1043	0.1021	0.0015
Average	0.9064	0.8234	0.8171	1.2947	1.5564	1.4102	0.1045	0.1089	0.0985	0.0015

**Table 9:** MAE obtained with a 20% of missing data MICE and new proposed AAA package.

	MAE MICE 20% MISSING DATA									
Iteration	Van	Vbn	Vcn	Vab	Vbc	Vca	Ia	Ib	Ic	PF
1	12.6252	25.2004	18.4560	33.2591	25.8328	35.2414	0.2007	0.2133	1.4431	0.0025
2	15.0349	26.6725	18.2779	28.8681	22.4685	34.9493	1.4675	0.2465	0.2272	0.0024
3	14.1887	20.7764	18.1093	36.6143	23.2577	36.0519	0.2438	0.2499	1.4036	0.0025
4	14.1482	25.9181	18.7435	28.1440	33.7646	37.7483	0.1893	0.2033	1.2435	0.0025
5	14.7270	25.3376	18.5203	28.8294	24.8967	38.1530	0.1971	0.2105	1.3253	0.0023
Average	14.1448	24.7810	18.4214	31.1430	26.0441	36.4288	0.4597	0.2247	1.1285	0.0024
	**MAE NEW ALGORITHM 20% MISSING DATA**									
**Iteration**	**Van**	**Vbn**	**Vcn**	**Vab**	**Vbc**	**Vca**	**Ia**	**Ib**	**Ic**	**PF**
1	0.7814	0.8120	0.8039	1.2398	1.3721	1.4579	0.1012	0.1155	0.0926	0.0014
2	0.8745	0.7664	0.8475	1.3372	1.5238	1.5322	0.1096	0.1105	0.0950	0.0015
3	0.9177	0.8772	0.8167	1.2134	1.3097	1.4525	0.1083	0.1145	0.0971	0.0014
4	0.8624	0.7406	0.7719	1.3691	1.4522	1.3876	0.1046	0.1086	0.1028	0.0015
5	0.8350	0.8022	0.8032	1.3666	1.3894	1.4925	0.1096	0.1062	0.0967	0.0015
Average	0.8542	0.7997	0.8086	1.3052	1.4094	1.4646	0.1067	0.1110	0.0969	0.0015

In the case of the metric MAE, the p-value was of p<0.001 for the kind of model in the variables ${\text{V}}_{\text{an}}$, ${\text{V}}_{\text{bn}}$, ${\text{V}}_{\text{cn}}$, ${\text{V}}_{\text{ab}}$, ${\text{V}}_{\text{bc}}$, ${\text{V}}_{\text{ca}}$, ${\text{I}}_{\text{b}}$ , ${\text{I}}_{\text{c}},$ and PF; for ${\text{I}}_{\text{a}}$ the p-value was of 0.038. Additionally, for the MAE metric, in the case of the variable percentage of missing data, there are no statistically significant differences between percentages (10%, 15% and 20%) obtaining the following p-values: 0.990 for ${\text{V}}_{\text{an}}$, 0.786 for ${\text{V}}_{\text{bn}}$ , 0.113 for ${\text{V}}_{\text{cn}}$, 0.887 for ${\text{V}}_{\text{ab}}$, 0.655 for ${\text{V}}_{\text{bc}}$ , 0.643 for ${\text{V}}_{\text{ca}}$ , 0.686 for ${\text{I}}_{\text{a}}$, 0.315 for ${\text{I}}_{\text{b}}$, 0.796 for ${\text{I}}_{\text{c}},$ and 0.424 for PF.

Finally, all the calculi of both the MICE and the AAA algorithm was performed with a computer equipped with an Intel Xeon E5-1650 processor and 16 GB RAM. The average time of the MICE algorithm runs was of 123.54 s. The AAA algorithm average completion time was of 74.36 s with a standard deviation of 8.32 s. In both case the dataset was formed by 17,763 samples each on them with 10 variables.

## 5. Conclusions

The existence of harmonics in electrical installations is an unavoidable issue nowadays. The use of real-time data collection devices is indispensable. During the process collection, it is possible for some data to be missing and, in this context, the use of missing data imputation techniques is essential.

The algorithm proposed in this research greatly improves the results obtained by means of one of the most renowned and common techniques used today. From the point of view of the authors, this new algorithm is of great interest for applications like the one proposed in the present paper. In spite of the good performance of the proposed algorithm, it must be also be taken into account that the proposed algorithm, like many others, would have imputation problems in those cases in which most of the missing data belonged to the same column or to a reduced subset of columns. In future research the use of support vector machines (SVM) [23,25] and hybrid methods [26,27,28] will be explored by the authors in order to find a new algorithm with even higher performance. Furthermore, authors will try to study the nonlinear time varying systems and other power quality features, taken into account proposals like [29,30]. Finally, another research line that will be explored is the missing data imputation in the time-frequency domain. It consists on estimating missing regions of the time-frequency representation of signals [31]. In this kind of researches, the imputation methods also make use of harmonics for the imputation, considering for instance, that in a certain moment there is missing information but that not all the information of all the frequencies is necessary lost at the same time. The algorithms developed would be of interest for any kind of signals.

The estimation of missing data is required in many different applications, such as time series analysis. The use of missing data imputation techniques allows the creation of prediction models using incomplete datasets.
